# International Issues in Immunization^1^

**DOI:** 10.3201/eid1011.040624_06

**Published:** 2004-11

**Authors:** Stephen L. Cochi, Lora Shimp, Francois Gasse, Jon K. Andrus

**Affiliations:** * Centers for Disease Control and Prevention, Atlanta, Georgia, USA;; † United States Agency for International Development/BASICS, Arlington, Virginia, USA;; ‡ United Nation's Children's Fund, New York, New York, USA;; § Pan American Health Organization, Washington, D.C., USA

**Keywords:** global immunization, immunization services delivery, maternal and neonatal tetanus, tetanus toxoid, rubella elimination, rubella vaccine

Women play a multidimensional role in the immunization enterprise: 1) as immunization program leaders and managers; 2) as vaccinators and community mobilizers; and 3) as recipients, together with their children, of immunization services. Women's roles in achieving Expanded Program on Immunization (EPI) immunization goals were presented.

## Women's Roles in Achieving Immunization Goals

The role of women is critically linked to immunization service delivery— as mother and community member ("gatekeeper," decision-maker), health provider (physician, nurse, community health agent, birth attendant, midwife, health educator), and as a target population for vaccination (e.g., women of childbearing age for tetanus toxoid) (*1*). As mothers and community members, women generally have the lead responsibility for bringing children for immunization services on a regular basis, according to the routine childhood immunization schedule, and in ensuring completion of the schedule. As health providers, women provide essential information and services for vaccination and liaise with communities to ensure compliance, acting as trusted female professionals and community members. As recipients of vaccines, women have the responsibility, not only for their own health, but, also that of their unborn children (e.g., rubella vaccine, tetanus toxoid). Women also play an important role in identifying and reporting on vaccine-preventable diseases as community-based informants. Immunization achievements could be further strengthened through improved communication and planning of immunization services with women and communities, collaboration with other women's health programs, increasing women's educational levels, building awareness and advocacy with women's groups and leaders, and promoting the use of user-friendly tools and approaches (e.g., vaccination cards, certificates) aimed at mothers as caregivers and target populations.

## Maternal and Neonatal Tetanus Elimination: Progress and Challenges

The World Health Organization (WHO), the United Nations Children's Fund, and United Nations Family Planning Agency are leading an initiative to eliminate neonatal tetanus as a major killer of newborns by 2005, with a current focus on 57 developing countries where most of the disease still occurs (*2*). Elimination is defined as reducing the incidence of neonatal tetanus to <1 case per 1,000 live births in every district of a country. Progress has been substantial: 787,000 neonatal deaths occurred in 1998, while the most recent estimate indicates a reduction to 180,000 deaths in 2002. Nonetheless, neonatal tetanus still accounts for 5% of all neonatal deaths. A major strategy for achieving neonatal tetanus elimination is to target women of childbearing age for vaccination with two doses of tetanus toxoid during supplemental immunization activities (mass vaccination campaigns). These efforts have accelerated in recent years, with the number of women of childbearing age increasing from 1.8 million women in 1999 to 33.8 million women in 2003. Despite this progress and the likelihood that 15 of 57 focus countries will have achieved elimination by 2005, 42 countries will require additional efforts, and resource constraints have emerged as a major obstacle to further progress.

## Rubella Elimination Initiative in the Americas

In the Americas, the Pan American Health Organization (PAHO) supports strategies to control and eliminate vaccine-preventable diseases, particularly when those strategies are designed to reduce inequities in health, strengthen political commitment for immunization, and foster the culture of prevention (*3*). The experience in the Americas demonstrates a longstanding history of soliciting and involving the support of mothers to protect children against vaccine-preventable diseases. PAHO strategies to eliminate diseases have always relied on vaccinating the susceptible population and conducting effective surveillance. By targeting communities with low vaccination coverage, women and children have been reached for vaccination who otherwise never would have received immunization services. Rubella elimination in the Americas offers new opportunities for increasing contacts of women with the health services, building bridges to draw attention to health needs of women, creating savings for the health system, and contributing to health infrastructure. The experience gained and lessons learned from vaccinating women of childbearing age will be useful for introducing new vaccines that will require adult vaccination strategies, such as those vaccines being developed for human papillomavirus and human immunodeficiency virus infections.

## References

[1] Shimp L. Women's roles in achieving immunization goals. Proceedings of the International Conference on Women and Infectious Diseases: from Science to Action, Atlanta, Georgia, USA, February 27–28, 2004. Atlanta: Centers for Disease Control and Prevention; 2004.

[2] Gasse F. Maternal and neonatal tetanus: progress and challenges. Proceedings of the International Conference on Women and Infectious Diseases: from Science to Action, Atlanta, Georgia, USA, February 27–28, 2004. Atlanta: Centers for Disease Control and Prevention; 2004.

[3] Andrus JK. Rubella elimination initiative in the Americas. Proceedings of the International Conference on Women and Infectious Diseases: from Science to Action, Atlanta, Georgia, USA, February 27–28, 2004. Atlanta: Centers for Disease Control and Prevention; 2004.

